# Exploring Convergence and Divergence in Seemingly Contrasting Perspectives on Training Perceptual-Cognitive Abilities for Sports Performance Through Moderated Dialogue

**DOI:** 10.1186/s40798-025-00904-y

**Published:** 2025-08-29

**Authors:** Jordan Cassidy, Daniel Kadlec, Job Fransen

**Affiliations:** 1 School of Exercise and Nutrition Sciences, Queensland University of Technology, Brisbane, Australia; 2 School of Medical and Health Sciences, Edith Cowan University, Joondalup, Australia; 3 School of Allied Health, Exercise and Sports Sciences, Charles Sturt University, Port Macquarie, NSW Australia

**Keywords:** Agility, Sidestepping, Adversarial collaboration, Ecological dynamics, Decision making

## Abstract

Current Opinion article considers the diverging perspectives of two academics on the trainability and role of perceptual-cognitive abilities in sports performance, specifically applied to agility and sidestepping. This work uses a moderated dialogue approach between these two authors, each representing differing viewpoints: one advocating for the role of perceptual-motor skills through representative learning environments and another emphasising physical resilience. The article explores how fostering scientific discourse through moderated questions posed by a third party can be used to identify convergences and divergences in these perspectives. Both perspectives agree on the complexity of agility, the value of coupling perceptual skills with motor actions in representative environments, and the role of action capabilities in shaping affordances. However, they diverge on the best methods for assessing and training these skills, with contrasting views on the practicality of representative assessments and training transfer to in-game scenarios. The authors propose that the current article forms the first stage for future collaborative research to test hypotheses through adversarial collaboration in order to better understand how perceptual-cognitive skills are integrated with physical training and assessed for practical application in sports settings. By fostering mutual understanding, the article highlights the potential of adversarial debate in advancing scientific practices within the domain of sports performance, as well as how this method can form the basis for joint hypothesis testing between adversaries.

## Key Points

• The dialogue revealed both areas of conceptual convergence and persistent divergence, particularly regarding the trainability of perceptual-cognitive skills and the design of representative training tasks.

• This exploratory process serves as a foundational step toward future adversarial collaboration, enabling the formulation of testable hypotheses and clarifying theoretical boundaries for subsequent empirical research.

• Practitioners are encouraged to recognise that improvements in agility may arise through both physical preparation and representative training design, and that neither approach alone may sufficiently prepare athletes for complex, in-game scenarios.

• Engaging in adversarial dialogue can be useful for developing new, testable hypotheses that can drive a research area forward. In this case, two testable hypotheses are proposed to guide future research: i) that incorporating task-representative perceptual-motor constraints to non-sport-specific sessions will enhance agility performance and decision-making compared to physical preparation training alone, and (ii) that representative, on-field agility assessments better predict in situ agility performance than decontextualised and standardised laboratory-based agility tests.

## Introduction

Perceptual-cognitive skills and/or abilities are widely recognised as integral to sports performance, often distinguishing elite from sub-elite athletes in their ability to anticipate, decide, and execute skills under varying conditions. Recent perspectives diverge on the role and methods for training these skills, particularly in agility and sidestep performance. Kadlec et al. (2023) [1] adopted a biomechanical lens focused on physical preparedness, proposing that increasing strength expands an athlete’s movement solution space, and critiquing the efficacy of perceptual-cognitive training for improving decision-making and anticipation, especially in non-representative settings due to limited transfer to on-field anticipation or decision-making. In contrast, Cassidy et al. (2024) [2] proposed the application of ecological dynamics, advocating for representative learning designs that couple perception and action to enhance adaptability in game-relevant contexts. This included an explicit endorsement of training perceptual-cognitive skills through constrained, sport-specific scenarios to promote transfer. Notably, Cassidy et al. (2024) [2] placed less emphasis on the physical underpinnings of movement capacity, instead directing attention toward the role of environmental interaction and task design in shaping skilled behaviour. While both authors have independently argued for evidence-informed approaches to developing agility, their respective emphases appeared to diverge substantially in prior work. These contrasting perspectives highlight the complexity and ongoing debate surrounding the most effective methods for training agility and sidestep performance, and the role played by perceptual-cognitive skills and/or abilities therein. As such, this article explores whether a moderated scientific debate could aid in aligning these seemingly contrasting perspectives.

Scientific discourse is crucial for the advancement of science, serving as a platform for collaboration, critique and the refinement of ideas. However, the scientific process itself can inadvertently foster strong attachments to personal perspectives, which can come at a cost of objectivity. For example, one’s tendency to interpret information that confirms one’s own perspectives is a commonly reported bias that befalls researchers (i.e. confirmation bias [3]). Additionally, echo chambers, where the loudest voices continuously echo the same perspectives, could create significant myopia in science especially under the influence of emerging new search technologies in scientific databases [4]. Actively engaging with those with different perspectives has been presented as a valuable approach to increase the validity of scientific work [5]. One such method is through adversarial collaboration where a team of scientists consisting of members deliberately chosen to represent diverse, contrasting and/or neutral perspectives work together to actively resolve disputes [6]. Most often, adversarial collaborations involve the identification of points of disagreement and then the mutual design of methods to test competing hypotheses in order to advance debate and generate reliable knowledge by restricting each party’s ability to rig methods in their favour [6]. While joint hypothesis testing is an important component of adversarial collaboration, adversarial collaboration often starts with the pre-registration of disagreements between adversaries, often moderated by a third person, in which researchers explore divergence or convergence of ideas, which can then form the basis for the subsequent development of a joint experiment to test competing hypotheses. As such, this article used a moderated dialogue between researchers who have previously written about their diverging perspectives on the importance and trainability of perceptual-cognitive abilities and/or skills in team sports settings in the context of the optimisation of agility and change-of-direction performance. It is the intent of the authors of this article to examine if moderated debate between their seemingly contrasting viewpoints on this topic leads to divergence or convergence of perspectives following moderated discussion. We intend this discourse to be pre-registered and submitted for publication to form the basis for the design of a research study to test competing hypotheses related to the role of perceptual-cognitive abilities and/or skills in sports performance.

## Methods

All components and stages of this article were pre-registered through the Open Science Framework (10.17605/OSF.IO/TSZ7R) for full transparency. All associated files have been made available through its project website on osf.io/eqrax.

This article uses a question-and-answer methodology moderated by a third, independent researcher to explore whether two seemingly contrasting perspectives on the importance and trainability of perceptual-cognitive abilities in team sports settings converge or diverge following a systematic process of moderated dialogue between the researchers with the contrasting perspectives.

In this manuscript, we define perceptual-cognitive abilities as the perceptual and/or cognitive processes underpinning decision-making and anticipation in response to dynamic stimuli in sport. We use the term *agility* to refer to reactive, context-sensitive movements requiring perception-action coupling, distinguishing it from *change of direction* (COD), which typically refers to pre-planned executed direction changes. Sidestepping is used as an exemplar movement due to its biomechanical complexity and relevance to injury-risk scenarios in field sports.

### Participants

The participants in this article were two authors of recently published scientific manuscripts with seemingly contrasting perspectives (JC, Cassidy et al., 2024 [2]; and DK, Kadlec et al., 2023 [1]. Both authors are currently employed at an academic institution and have both practical and academic experience related to the training of perceptual-cognitive abilities in the context of sports performance.

### Procedure

This project was pre-registered on Open Science Framework on 21/09/2024 upon which DK and JC were invited by the third author (JF) to respond to pre-moderated questions generated by JF upon reading the relevant manuscripts by DK and JC [1, 2]. The authors were encouraged to write down responses to five questions (see Table 1) using 400 words or less within a four-week time span. The time and word limits were provided so responses would reflect the authors’ perspectives in a clear and concise manner. Following the submission of their first responses to the questions, the responses were collated and uploaded to the Open Science Framework, before the authors were invited to modify their responses based on each author’s entries, which were provided to both authors in correspondence sent to both authors on 15/10/2024. Both authors were given two weeks to modify their responses and were limited to a final response of no more than 500 words per question. Finally, the authors were sent the collated responses on 28/10/2024 upon which they were invited to consider in a discussion section their reflections upon the convergence or divergence of their perspectives following this systematic process.

**Table 1:** Moderated questions based on Cassidy et al. (2024) [2] and Kadlec et al. (2023) [1]

Q1	What is the core theoretical and/or practical framework that shapes your perspective on the role or importance of perceptual-cognitive abilities and/or skills in the performance of sports-related motor skills such as agility or side-stepping? Highlight its key tenets.
Q2	Given you both emphasise the role of perceptual-cognitive abilities and/or skills in the performance of sports-related skills, to what extent are perceptual-cognitive skills trainable, and how does this shape your perspective on the role or importance of perceptual-cognitive abilities in the performance of sports-related motor skills such as agility or side-stepping?
Q3	To what extent should the development of perceptual-cognitive abilities and/skills be integrated with physical training to optimise the performance of sports-related motor skills such as agility and sidestepping?
Q4	Considering your perspective, what would be the most effective methods for assessing perceptual-cognitive abilities and/or skills in agility and sidestepping, and how do these assessments inform the design of training interventions in practical settings?
Q5	How do you ensure the transfer of perceptual-cognitive training to real-world sports performance such as the performance of agility or side-stepping in competition, and what are the strengths and/or limitations proposed in your respective perspectives related to achieving this transfer?

The analysis and interpretation used in this manuscript is entirely subjective and based first and foremost on the experiential knowledge and scientific perspectives of the moderator upon reading [1, 2] the experiential knowledge and scientific perspectives of JC and DK in responding to the moderator’s questions and on the collaboration between JC and DK in examining and discussing convergence or divergence in the authors’ responses to the moderated questions.

## Results

This results section outlines the responses to each of the questions for DK and JC. The original responses, modified responses after review and moderation letters can all be found at osf.io/eqrax.

### What Is the Core Theoretical and/or Practical Framework that Shapes Your Perspective on the Role or Importance of Perceptual-Cognitive Abilities and/or Skills in the Performance of Sports-Related Motor Skills Such as Agility or Side-Stepping? Highlight its Key Tenets

#### DK

Perceptual-cognitive abilities and/or skills are typically considered to be essential for success in team sports. The process of perceiving, interpreting, and integrating contextual information, often referred to as “reading” the game, is widely considered a critical aspect of agility performance [7]. While it is important to distinguish between perceptual-cognitive abilities and skills, among sports practitioners and athletes, it is often assumed that superior perceptual-cognitive skills and/or abilities leads to faster, more accurate anticipation and decision-making on the field. While perceptual-cognitive skills and/or abilities are important, their role in sports performance may be more complex than often reported. The following arguments define my practical framework as to the role and importance of perceptual-cognitive skills and/or abilities as a (strength & conditioning [S&C]) practitioner.

Cross-sectional research suggests that elite athletes generally outperform sub-elite athletes on perceptual-cognitive tasks like response accuracy and response time with representative stimuli [8]. However, comparing different groups only at a single point in time may limit our understanding of cause and effect [9] and intervention or longitudinal studies in this domain rarely follow methodological recommendations from established frameworks [10]. Further, the wide variability in anticipation and decision-making skills, even among elite team-sport athletes, suggests that the execution of perceptual-cognitive skills and/or abilities tasks is highly individual and may not always be well-reflected in group-averaged data [11]. The lack of quality evidence inherently limits our understanding of the role of perceptual-cognitive skills and/or abilities in sports performance [12].

Next, it is important to recognise that perceptual-cognitive skills and/or abilities represent only one sports performance sub-component. Success in team sports predominately relies on exploiting the non-linear and interdependent interaction from physical, technical, social, and perceptual-cognitive factors. Each sub-component influences the others, creating a complex dynamic system [13]. Superior decision-making may not improve performance, even in sports-specific agility tasks, unless supported by sufficient physical capacity and adequate technical proficiency within goal-oriented team behaviour [14–16].

**Fig. 1:**
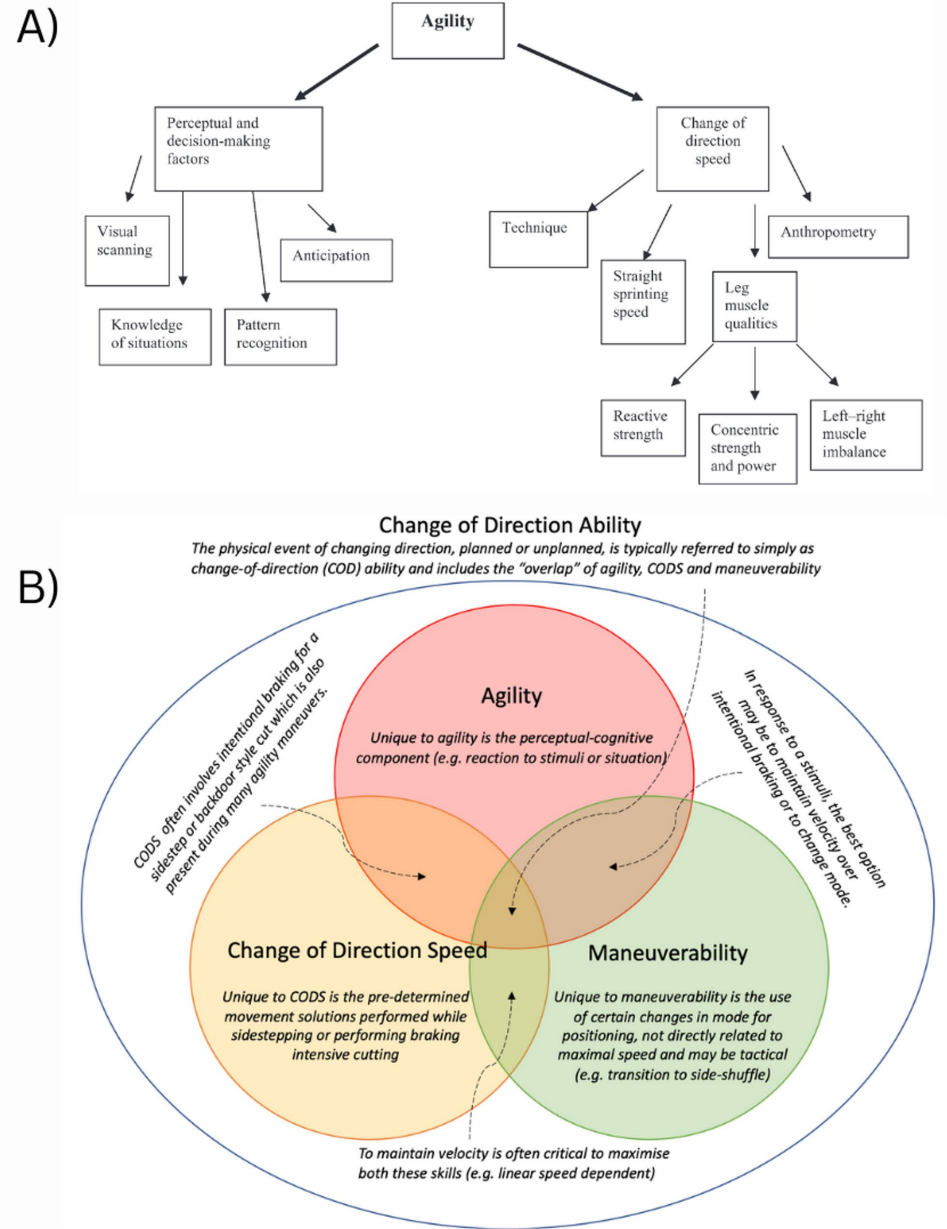
**(A)** Universal agility components. Reproduced from Sheppard and Young [18], with permission. **(B)** Overarching change of direction ability: A Venn diagram of agility, change of direction speed and maneuverability [19]. CODS = Change of direction speed

Finally, current definitions of agility are typically presented as hierarchical models and isolate components like perceptual and cognitive factors and change of direction speed with all their sub-components, implying a causal relationship (Fig. 1A). These models prioritise measurable aspects like a change of direction speed while neglecting the interdependent and non-linear nature of agility tasks. Due to these complex interactions, operationalising agility as a singular, quantifiable measure remains a methodological challenge in sports science [17]. Consequently, current models and definitions of agility may introduce bias in how we understand, research and train for agility performance. A more holistic approach to defining agility performance may improve research quality and training recommendations (Fig. 1B).

Although perceptual-cognitive skills and/or abilities training could hypothetically enhance decision-making and anticipation, their transfer to in-game performance remains questionable and warrants more empirical research adhering to robust research practices. The lack of quality evidence, the perceptual-cognitive skills and/or abilities role as just one component of a holistic performance system, and the limitations of current definitions highlight avenues for future research. With these limitations and the non-trivial uncertainty around the role and importance of perceptual-cognitive skills and/or abilities, it is challenging to prioritise training perceptual-cognitive skills and/or abilities over other areas as a (S&C) practitioner.

#### JC

The core theoretical framework that informs my perspective on agility is ecological dynamics (ED), which is a meta-theoretical framework consisting of ecological psychology, dynamical systems theory, and complexity science [20, 21]. Through an ED lens, the athlete-environment relationship becomes the basis of understanding sports performance and development. Regarding agility, which can be defined as a complex skill involving the coupling of perceptual information to guide actions, and vice-versa [2], it is therefore not enough to consider an athlete’s movement in isolation. Rather, this movement must be considered in relation to the athlete’s environment. Chow et al., (2019) [22] identified four key principles of ED.

- **Information-movement coupling**: information regulates action, and action regulates information pick-up. This continuous cycle is the basis of learning under an ED approach [20]. By placing athletes within a dynamic training environment, they will increasingly become more attuned to critical information within their environments and, thus, learn to perceive relevant information and adapt their actions.
- **Affordances**: affordances can be described as invitations for actions, always situated within the environment [23]. The theory of affordances is central to ecological dynamics [24]. Affordances can be defined as opportunities for action [25]. An affordance presented by the environment does not have to be acted on by an athlete, but it can be utilised by an athlete with specific capabilities; i.e. affordances are athlete specific and can be body-scaled or action-scaled, meaning they are defined relative to the action capabilities of an athlete [25, 26].
- **Representative learning design**: The underpinning rationale of representative learning design (RLD) is that transfer from training to competition is more likely to occur when the conditions in the training environment replicate the conditions in the competition environment [26]. If the information present during a training task is similar to the information present in competition, athletes can learn to use perceptual information to help regulate their actions [27].
- **Manipulation of task constraint**: The constraints-led approach [28] is considered a pedagogical approach to support skill development through ED theory. For coaches, manipulating task constraints in practice (i.e. practice design) can make affordances more or less relevant or inviting for an athlete to act on.

### Given You Both Emphasise the Role of Perceptual-Cognitive Abilities and/or Skills in the Performance of Sports-Related Skills, to What Extent Are Perceptual-Cognitive Skills Trainable, and How Does This Shape Your Perspective on the Role or Importance of Perceptual-Cognitive Abilities in the Performance of Sports-Related Motor Skills Such as Agility or Side-Stepping?

#### DK

Whether perceptual-cognitive skills and/or abilities are trainable requires more context to provide actionable recommendations. Improvements can range on a continuum from reactions to isolated, generic stimuli with verbal or micro-action responses targeting lower-order perceptual-cognitive skills and/or abilities to complex, sport-specific stimuli with adequate motor responses to improve decision-making and anticipation [10]. While lower-order perceptual-cognitive skills and/or abilities, such as reaction time and visual scanning, can be improved through isolated training, the more exciting yet challenging question is how training perceptual-cognitive skills and/or abilities can transfer to in-game performance. The current evidence does not allow us to answer this with meaningful certainty [29].

Improving lower-order perceptual-cognitive skills and/or abilities (e.g., reaction time, visual scanning and gaze control) shows promising results in controlled environments with specific training [30]. However, traditional perceptual-cognitive skills and/or abilities training methods often involve abstract stimuli (e.g., flashing lights) and simple verbal or micro-movement responses. While these tasks improve lower-order perceptual-cognitive skills and/or abilities, the lack of stimulus and response correspondence to sport-specific motor actions limits their transferability to in-game scenarios [31].

In contrast, higher-order cognitive skills, such as sports-specific decision-making and anticipation, require more representative stimuli and more specific motor actions to potentially improve in-game performance [10, 12, 32]. These higher-order skills involve complex executive functions like working memory, cognitive flexibility, and pattern recognition, which are reported to be crucial for making rapid, adaptive decisions in unpredictable situations [33].

Although many studies report near-transfer effects, where improvements in reaction time or decision-making are observed within the context of the training task, it is unclear whether these gains lead to far-transfer and result in enhancements of in-game performance [10]. While it is plausible that a series of near-transfer gains could collectively improve game performance over time, the argument supporting this process remains currently speculative [34].

Moreover, many perceptual-cognitive skills and/or abilities training studies face limitations, including (i) reliance on computer-based tests that fail to mimic game conditions, (ii) assessments that isolate perceptual-cognitive skills without integrating them with sport-based movement responses, and (iii) a lack of evidence for the reliability and validity of the assessment tasks themselves [10, 29]. Without clear evidence of test reliability, discriminative validity (i.e., showing performance differences based on the athlete’s level), or accuracy in measuring these improvements, there is limited certainty that training isolated perceptual-cognitive skills and/or abilities will transfer to in-game performance. Given these uncertainties, practitioners need to carefully evaluate how to design and implement perceptual-cognitive skills and/or abilities drills and exercises to improve in-game anticipation and decision-making.

One indirect approach to improving anticipation and decision-making indirectly is by increasing athletes’ action capabilities (e.g., sprint speed or jump performance). Athletes with superior action capabilities are afforded the ability to wait longer before initiating movements, allowing them to perceive more reliable information longer and thus improve decision accuracy [15, 35]. Conversely, weaker athletes are forced to initiate movements earlier, potentially gambling with their choices. As such, insufficient action capabilities or the underpinning physical capacities (e.g., strength and power), can therefore act as a constraint on perceptual-motor skills and limit the athlete’s ability to execute decisions timely and accurately [36, 37]. This implies that complementary training approaches of both perceptual-cognitive skills and/or abilities and action capabilities seem beneficial to prepare athletes for in-game demands.

#### JC

Given that an athlete’s action capabilities are always developing due to short-term factors like fatigue, or long-term factors, like injury, actions can become more or less possible [38]. Development can include changes in an athlete’s body (e.g., size and strength of limbs) and an athlete’s movement capacities (e.g., greater force production), which opens or closes new affordances [39]. Therefore, development can re-shape the field of affordances of an athlete. As an athlete’s field of affordances is always changing, athletes continuously need to calibrate and re-calibrate to their action capabilities [40]. The process of calibration involves an athlete scaling between their current action capabilities and the current task [20].

In relation to agility, increased force production may mean that a gap that was once insufficient to accelerate through now presents an affordance, or vice-versa. This re-calibration is an example of how perceptual cognitive skills are trainable throughout an athlete’s career. Adolph (2019) refers to this as a process of learning – what an athlete must do to cope with the changing field of affordances. Therefore, the enhancement of perceptual-cognitive skills can be reframed as a learning in development process, whereby athletes acquire the real-time flexibility to guide their actions in a dynamic environment. This illustrates the importance of including representative information in training tasks so athletes can learn to use appropriate perceptual information to guide their movement behaviour. Recalibration may occur quite quickly but given that further experience in each task can lead to further calibration improvements, and that an athlete is developing on both long and short timescales, the development of agility performance may require regular exposure to representative tasks, where athletes “learn to generate and detect information for affordances at each moment – what they can do with this body and these skills in this environment for this task.” (Adolph, 2019, p. 189) [39].

However, given that I have framed the trainability of perceptual-cognitive skills as a process of learning in development, where learning and development are parallel processes, it also highlights the role strength and conditioning can play in developing agile movers. The development of muscular strength and power and intramuscular coordination, even in isolated and decontextualised conditions relative to the competition environment, can support an athlete in perceiving and acting on affordances that were previously impervious, provided the athlete is being exposed to enough representative practices.

### To What Extent Should the Development of Perceptual-Cognitive Abilities and/or skills Be Integrated with Physical Training to Optimise the Performance of Sports-Related Motor Skills Such as Agility and Sidestepping?

#### DK

Integrating perceptual-cognitive skills and/or abilities with physical training, whether within S&C or sport-specific practice, serves different purposes. It is crucial to understand both the benefits and limitations of each approach.

The affordance-based control hypothesis suggests that athletes’ decision-making and anticipation are constrained by their action capabilities [14, 25]. In simple terms, athletes perceive and act on the possibilities that align with their physical capacities, such as strength, speed, or power [37, 41]. Any changes in these physical capacities, whether due to training adaptations or fatigue, can affect how athletes perceive and exploit information related to affordances and how they execute perceptual-motor skills (e.g., agility tasks or sidesteps) [42, 43]. Although athletes may be perceptually attuned and have the capability to act, successful performance requires them to calibrate their actions to perceptual information and vice versa. This calibration process involves learning how to effectively utilise their physical capacities and can be facilitated through training [44].

In S&C practice, the primary focus is improving physical preparedness by increasing an athlete’s physical capacity (e.g., strength, speed, power). As these capacities evolve, S&C practitioners can design drills and exercises to provide athletes with opportunities to iteratively calibrate their action capabilities, transforming them into effective and efficient perceptual-motor actions under varied conditions [1]. S&C practitioners can introduce complex decision-making tasks, such as during agility drills with varying levels of stimuli complexity or reaction times, to facilitate athletes’ calibration of action capabilities. Different stimuli (e.g., visual or auditory cues, varying opponent movements) afford different preparation times, forcing athletes to perform tasks like sidesteps under varied conditions and demands [45, 46]. This variation helps athletes adjust their action capabilities to the information available and the context, making them more adaptable to in-game situations.

Additionally, such drills build robustness and resilience against injuries when progressively designed and implemented. Although these drills do not directly improve in-game decision-making, they prepare athletes for unpredictable, high-pressure scenarios by tolerating the imposing physical demands [1]. While exposing athletes to varied drills across different environmental contexts to facilitate the calibration of action capabilities is an appealing approach, the unpredictable nature of in-game scenarios presents a meaningful challenge. Given the potentially infinite scenarios athletes may encounter in games, attempting to prepare them for every possible situation becomes impractical and impossible. Instead, S&C coaches could prioritise perceptual-motor skills closely related to key performance outcomes or high injury-risk tasks (e.g., sidesteps). By focusing on these potentially performance-determining yet high-impact skills, S&C practitioners can ensure that athletes are better prepared for these crucial scenarios of competition, leaving more sports-specific perceptual-cognitive adaptations to be refined in sport-specific training sessions. As such, sports-specific practice may provide a more representative setting for integrating decision-making, anticipation, and team-based strategies, affording greater opportunities to enhance competitive success [47].

In summary, the role of S&C in developing perceptual-cognitive skills and/or abilities skills is not to directly improve decision-making or anticipation but to create drills and exercises that facilitate the calibration of action capacities to various and game-relevant perceptual-motor skills (e.g., sidesteps), while increasing physical robustness and resiliency.

#### JC

From the perspective of developing perceptual-cognitive skills, it is important to incorporate physical demands into practice, to ensure conditions in practice are representative of the conditions in competition, particularly the physical demands (i.e. representative learning design). This can ensure athletes experience performing under fatigue and can challenge athletes to perform under similar time constraints. Utilising concepts like the game-intensity index [22], affective learning design [48] and the task design model [49] can support coaches to include representative information in practice to ensure the physical demands of practice are comparable to competition.

However, it is also important to consider an individual’s capabilities, the functional difficulty of a task, and the individual variability of an athlete’s response to such challenge [50]. It is also important to recognise that every practice or training task does not require the presence of representative information, as this could lead to a risk that athletes do not fulfil their physical potential. Decontextualised change of direction drills (i.e., practice tasks that lack representativeness) can be more useful to develop the physical capacities of athletes in comparison to agility activities [51]. A useful systematic review to highlight that training in high perceptual-cognitive demands may not create enough of a stimulus to support physical development is Hill-Haas et al. (2011) [52]. It was suggested that athletes with a good skill level and an already high fitness level will not exercise at a sufficient intensity to stimulate aerobic adaptations. Skillful athletes and teams will adapt their playing strategy based on their fatigue levels. While this is an example of good perceptual-cognitive skills, it will not stimulate physical development, which could ultimately narrow the field of affordances of an athlete.

Therefore, it should be noted that what is optimal for the development of perceptual-cognitive skills may not be optimal for the development of physiological, psychological or biomechanical capacities, which are all equally important for coaches to consider, to ensure the holistic development of their athletes. This question incorporates two key knowledge bases of a coach – knowledge of skill acquisition, and knowledge of the ‘ologies’, specifically physiology [53]. Depending on their own context, coaches need to be adaptable when addressing such performance problems [54–57].

### Considering your Perspective, What Would Be the Most Effective Methods for Assessing Perceptual-Cognitive Abilities and/or Skills in Agility and Sidestepping, and How Do These Assessments Inform the Design of Training Interventions in Practical Settings?

#### DK

The goal of any assessment is to inform training-related decision-making. The practical relevance of assessing lower- and higher-order perceptual-cognitive skills and/or abilities lies in their potential to quantify performance-limiting factors within agility and sidestepping tasks and guide individualised training interventions. However, the challenge is ensuring that the type of test and outcome metrics are ‘generalisable enough’ to account for the extensive degrees of freedom inherent during in-game agility performance and provide practitioners with actionable recommendations.

A central issue of testing perceptual-cognitive skills and/or abilities for agility performance is the trade-off between standardisation and ecological validity. Most tests evaluating reaction or decision-making time benefit from controlled, repeatable conditions but often lack the ecological validity needed to account for the complexity of in-game agility tasks. Increasing ecological validity by making tests more representative of in-game situations, by ensuring high stimulus and response correspondence [10], may reduce the reliability of testing procedures and outcome metrics [58]. In-game agility performance relies on ongoing and cyclical perception-action processes, where athletes perceive, interpret, integrate, and execute perceptual-motor skills based on contextual information [24]. Even lab-based agility tests that incorporate more realistic scenarios (e.g., on-screen footage of opponents or virtual reality environments) may fail to fully replicate the contextual demands of in-game tasks [59, 60]. Especially since lab-based tests rely on passive stimuli, the motor response lacks any consequences or interaction with the decision made, inviting athletes to “gamble” their responses. Without these consequences, any inferences about how or if perceptual-cognitive skills and/or abilities limit in-game performance remain questionable. Moreover, many lab-based perceptual-cognitive or perceptual-motor assessments may evaluate a skillset that may not fully replicate the demands of in-game scenarios [61]. As a result, athletes who perform well in controlled tests may not necessarily exhibit the same levels of perceptual-motor effectiveness and decision-making accuracy during in-game scenarios. The complexity of assessing performance-limiting factors during agility tasks remains an ongoing methodological challenge.

Given these limitations, the subjective evaluation of sports coaches about athletes’ in-game performance may offer a practical alternative for assessing perceptual-cognitive skills and/or abilities. With their extensive experience and continuous observation of athletes in games, sports coaches may better gauge athletes’ perceptual-motor skills, including their decision-making and anticipation skills, than isolated lab tests with limited generalisability [62]. Unlike lab tests, coaches’ evaluations include emotional, psychological, and environmental factors, offering a broader view of how athletes respond within sports-specific situations. This may provide practitioners with a pragmatic solution for evaluating an athlete’s perceptual-cognitive skills and/or abilities. However, such subjective evaluations may introduce bias due to personal interpretations or preconceived notions, potentially overlooking individual differences or favouring certain athletes.

While testing isolated perceptual-cognitive skills and/or abilities holds theoretical potential, using these test results to inform training decisions remains challenging. The lack of quality evidence demonstrating that improvements in perceptual-cognitive skills and/or abilities test outcomes translate to in-game decision-making and anticipation makes it difficult to draw actionable recommendations. To inform practice, assessing perceptual-cognitive skills and/or abilities must reliably discriminate between cohort levels, demonstrate that targeted perceptual-cognitive skills and/or abilities are trainable, and show that improvements transfer to in-game performance [10]. Until these conditions are met, sports coaches’ evaluations may offer more useful insights to inform training.

#### JC

Given that athlete’s perceive affordances based on the information they pick up in the environment and their own action capabilities, this therefore indicates that any assessment of perceptual-cognitive skills should aim to assess an athlete’s action in an environment that is representative of the environment they perform in. Therefore, competition, or tasks that are representative of competition where athletes demonstrate their ability to act based on information pick-up in their environment, is best for assessing perceptual-cognitive skills. Practitioners can then adopt an approach of “testing is training, training is testing” and monitor the development of perceptual-cognitive skills throughout a competitive season.

Representative assessments allow for coordinated and collaborative training interventions. For example:

- An AFL coach and performance analyst identify that athlete A is particularly strong performing attacking agility maneuvers when her back is to goal but struggles when in a face-to-face scenario. Athlete A is a half-back, her attacking agility actions are predominantly in a face-to-face position, so this becomes a key work-on for the athlete. The head coach, performance analyst and athlete all sit down to discuss this and during individual time after training, the coach and athlete co-design representative tasks to improve this aspect of play [63]. The performance analyst will monitor closely this attacking agility scenario for the next six weeks along with collecting qualitative feedback on how the athlete feels while performing.
- A football (soccer) coach has recognised that while his team is performing well in attacking and defensive organisation, they struggle to execute well in transition [64]. Through discussion with his assistant coaches, they identify that while many players are strong in the build-up play, and connect for a strong defensive block, all players are struggling in their small-sided duels (1v1, 2v1, 3v2 etc.), in attack and defence. To gain further insight, they asked the leadership team for their perspective, and collectively it was identified that roughly 80% of their training was medium- to large-sided games (6v6 and up). To develop performance in these troubling situations, coaches implemented a 2v2 + 1 recovering defender, where all players were exposed to all roles through frequent rotations to mimic the chaos of transitions. The sequencing of this activity varied from early in the session post warm-up, to last thing to ensure athletes are required to perform transitions under pressure. Coaches continued to monitor performance in representative tasks in practice and competition to evaluate change.

### How Do You Ensure the Transfer of Perceptual-Cognitive Training to Real-World Sports Performance Such as the Performance of Agility or Side-Stepping in Competition, and What Are the Strengths and/or Limitations Proposed in Your Respective Perspectives Related to Achieving this Transfer?

#### DK

Designing training to ensure the transfer of learned skills into in-game performance is a crucial but challenging process and requires adherence to several key principles. To maximise transfer, training needs to create practice environments that closely replicate the physical, perceptual, and cognitive demands of competition, ensuring athletes are exposed to stimuli they will encounter in games. Additionally, the perceptual-motor skills executed during training are required to match those in competition. The affective learning design framework further highlights the importance of integrating emotional and psychological stressors into training to simulate the pressures athletes face in competition [48]. Fulfilling these requirements is difficult, even within sport-specific training, and becomes more challenging in S&C practice. This raises important questions about how to efficiently allocate resources to maximise player development and long-term success. In other words, popular drills often used within S&C practice, such as different progressions of 1v1s with various task complexities, can only be considered a general activity with, at best, a trivial transfer potential to in-game anticipation and decision-making.

The proposed approach to ensure transfer is to primarily focus on building physical robustness and resilience, especially during S&C practice, by progressively exposing athletes to high-impact activities (e.g., sidesteps) and varied scenarios. This allows athletes to ongoingly calibrate their action capacities into effective perceptual-motor skills for the unpredictable and demanding competition environments [14]. By enhancing their capability to withstand and adapt to the repeated physical demands of their sport, or in other words, to be able to “succeed despite”, athletes can engage and experience more consistent, sport-specific practice and competition. This consistency affords athletes the opportunity to accumulate valuable exposure to competition-specific perceptual and cognitive demands that are difficult to replicate in practice or isolation. Despite growing interest in enhancing perceptual-cognitive skills and/or abilities, accumulating deliberate practice seems key for sport-specific mastery. Athletes who consistently engage in practice can refine perceptual-cognitive and perceptual-motor skills in sports-specific environments. Rather than attempting to accelerate perceptual and cognitive development through questionable approaches, the primary focus should be ensuring athletes remain robust and resilient enough to consistently train and compete.

The key strength of this approach is its reliance on and acknowledgment of the efficacy of traditional or “time-tested” training methods, considering the limited time available for S&C practice [65]. This viewpoint allows the confident omission of unproven and potentially pseudoscientific methods, which are often appealing to sports coaches in search of marginal gains [66]. By adhering to mundane yet effective training methods, the focus remains on building athletes’ robustness and resilience and preparing them for the imposing demands of the sport. This pragmatic approach allows for efficient time use, a critical argument considering the frequent lack of physical preparation opportunities, particularly for adolescent women athletes [67].

One potential limitation in predominantly relying on traditional training approaches is that athletes either adapt and thrive or fail to progress. This “survival of the fittest” model may disadvantage athletes who could benefit from the process of incorporating tools and methods to improve their perceptual-cognitive skills and/or abilities despite the current lack of quality evidence.

#### JC

The essence of transfer is being able to adapt a pre-existing movement pattern to a different context [68]. Under an ecological dynamics perspective, it is the coupling of movements to information that needs to transfer from practice to competition [26, 27]. In ecological dynamics, transfer occurs on a specific to general continuum [22], and specificity of transfer is facilitated by the representativeness of a training task, i.e., representative learning design [27]. In the same way representative learning design is used in testing perceptual-cognitive skills, it should be used when training these skills – further adding weight to the “testing is training, training is testing” approach. Here, recognising the key informational variables, like the presence of defenders [69], scoreboard pressure [70], and a direction of play [49] are all examples of informational variables that learners use to appropriately regulate their actions. When considering transfer from practice tasks to game performance to occur, a useful model to consider is Hodges and Lohse (2022) [50] metatheoretical challenge-based framework model, where representativeness is considered along with an individual’s challenge point to design “practice to transfer” type activities.

While ecological dynamics presents an attractive framework for coaches when considering the transfer of perceptual-cognitive skills and ensures that athletes are preparing for their competitive environment, it is also difficult to measure or track quantitatively. With representativeness comes variability, meaning that every agility maneuver in representative conditions will be different, making it challenging to compare one movement to another and track progress over time. Alternatively, practitioners can control the environment to such an extent that contextual variability is limited or non-existent to allow for replicability, allowing athletes to complete a pre-planned or a reactive agility step with limited options [71]. However, this approach does not adhere to the aforementioned ecological dynamics principles [22]. Therefore, it is challenging to test for causality when rigidly applying an ecological dynamics approach.

While general tasks (like traditional strength and conditioning testing and training) may lack representativeness, it has long been acknowledged that they can provide a foundation to enhance agility, balance, coordination and proprioceptive abilities [72], and crucially, they can be tracked and monitored in relatively stable environments over time, presenting coaches with more concrete data over time to monitor their athletes’ developmental pathway. Therefore, the combination of specific (representative tasks in practice) and general (strength and conditioning practices) learning will likely lead to the optimal development of agility.

## Discussion

The following discussion uses the responses to moderated questions about authors’ perspectives on the role of perceptual-cognitive skills during agility tasks (including sidesteps) from two authors who previously published contrasting perspectives to examine whether a moderated discussion could cause a convergence or divergence of their perspectives. Convergence in this context refers to both authors’ alignment on all key arguments and justifications provided, while divergence represents differences in the interpretation or implementation of the role and importance of perceptual-cognitive skills. Complementarity, in contrast, implies that both viewpoints offer mutually enhancing perspectives, even if independently distinct.

For **Q1**, both perspectives complementarily highlighted the complexity of agility tasks, which is currently not adequately captured with traditional definitions and, thus, proposed the meta-theoretical framework of ecological dynamics to better understand the emergence of agility performance. Both perspectives converged on the view that perceptual-cognitive skills influence agility performance but only during the coupled execution of perceptual-motor agility tasks within representative environments. As such, understanding and training perceptual-cognitive skills which are decoupled or isolated from their performance environment may not impact the development of agility performance in competitive settings.

For **Q2**, on the trainability of perceptual-cognitive skills, both adversaries complementarily agreed with each other’s arguments, especially on the impact of an athlete’s action capacities (e.g., sprint speed or jump performance) or the underpinning physical capacities (e.g. muscular strength and power) can have on the field of affordances and thus on the execution of perceptual-motor skills. Both viewpoints ultimately led to convergence of perspectives.

Both adversaries converged on the argument about the extent of integrating the development of perceptual-cognitive skills with physical training in **Q3**. Specifically, they aligned on the fact that non-representative and decontextualised practice is an effective method to develop the underpinning physical capacities and complementarily viewed that representative training may be less effective to overload physical capacities. Isolated individual drills within S&C sessions, although limited in the representative value, are still of value to enhance physical capacities, which will positively impact an athlete’s field of affordances. Hence, the answer to the question: “To what extent should perceptual-cognitive skills and/or abilities skills be integrated with physical training?” is that it depends on the aim. It may be possible to stimulate aspects of the representative environment with concepts like the game-intensity index, affective learning design, and the task design model outside of sports-specific practice (i.e. within separate S&C sessions). However, the effectiveness of such an approach for improving in-game perceptual-motor skills warrants further research. Both adversaries converged on the view that sports-specific practice may likely provide the most representative and, thus, effective setting to develop sports-specific decision-making and anticipation.

One point of divergence in **Q4** centred on the most effective approach for assessing perceptual-cognitive skills. The question about the efficacy of the representative assessments based on in-game performance and the feasibility of isolating, replicating, and ultimately improving these specific in-game scenarios previously identified as performance-limiting remained a point of contention and divergence between the authors.

For **Q5** and how to ensure a transfer of perceptual-cognitive training to real-world sports performance of agility tasks both perspectives highlighted the need to train in representative environments to match the complex in-game demands. Additionally, both converged on the need for and the impact of building physical capacities with either general and unspecific training methods (e.g. resistance training) and decontextualised exposure to perceptual-motor skills (e.g. preplanned sidesteps) to develop agility performance. An ongoing challenge for practitioners is knowing when to prioritise specific, representative exposure, and when to prioritise general physical capacity development to promote transfer from training to in-game scenarios.

Overall, this moderated discussion demonstrated a strong convergence in perspectives on the role and importance of perceptual-cognitive skills for sports performance, with both adversaries complementarily aligning more closely than in their original articles. Only minor points of divergence remained, specifically regarding sub-arguments in two questions, where differing perspectives on assessment and training settings persisted. This demonstrates that (1) what were originally considered contrasting perspectives by two authors of these perspectives are, in reality, far more convergent views on the role played by perceptual-cognitive skills and abilities in the context of the training of sports performance, (2) it may be valuable for those engaging in team science to deliberately seek out those with differing perspectives to one’s own without therefore compromising the scientific integrity of each party’s perspectives, and (3) moderated discussion may be interesting to find points of convergence and divergence between seemingly different perspectives in sport and exercise.

These moderated questions exemplify the next step of adversarial collaboration: moving beyond dialogue to joint hypothesis formulation, experimental design, and empirical testing. Rather than resolving debates through consensus alone, adversarial collaboration offers a mechanism by which ideas are clarified, made testable, and, critically, allowed to fail. In such a setting, weak or inconsistent ideas are more likely to be exposed and discarded, while promising concepts become more refined through iterative challenge. This process not only accelerates theoretical clarity between competing perspectives but also reduces ambiguity and contradiction in the published literature, thereby equipping future researchers to generate more focused, productive, and testable hypotheses.

This work demonstrates the feasibility and conceptual utility of using a moderated dialogue format to initiate structured theoretical exchange in sport science. The format enables transparency in articulating assumptions, clarifying terminology, and exploring the contextual dependencies that often underlie disagreement in applied fields. The process provides a nuanced record of how theoretical frameworks are interpreted and operationalised in practice by capturing detailed reasoning from both adversaries. However, limitations include the inherent subjectivity of dialogue-based information and the interpretive nature of convergence analysis.

## Conclusion and Future Directions

Given that it is now clear where areas of dispute remain between the two adversaries in this article, these differing viewpoints can be used to set up an adversarial collaboration through the mutual design of research methods to test both adversaries’ competing hypotheses [6]. Two points of contention, or divergence, remain between the adversaries, which could form the foundation of a subsequent intervention study to be designed collaboratively between the two adversarial parties. First, can concepts such as game-intensity indices, affective learning design, and task design models outside of sports-specific practice (i.e. within separate S&C sessions) meaningfully train perceptual-motor skill improvements and improve in-game perceptual-motor skills, or are sports-specific practices the most effective setting for developing decision-making and anticipation? Second, do representative assessments, or assessments in controlled environments, offer a valid and reliable assessment tool to assess the perceptual-cognitive demands of sports performance?

Each of these divergences can be formalised into specific, testable hypotheses to guide future empirical research designed collaboratively. For example, (i) an intervention delivered within the non-sport-specific on-field sessions (i.e. within separate S&C sessions) that incorporates task-representative perceptual-motor constraints (e.g., affective learning design, and decision-based agility drills) will result in significantly greater improvements in in situ agility performance and anticipatory decision-making compared to an on-field physical preparation program without perceptual-motor elements; and (ii) representative agility assessments with high ecological validity (e.g., on-field 1v1 decision-making scenarios) will demonstrate stronger concurrent validity with in-game agility performance metrics (e.g., successful evasive manoeuvres, decision quality ratings) compared to standardised, decontextualised and laboratory-based agility tests. These hypotheses serve as a conceptual starting point. Translating them into robust experimental designs will require careful specification of reliable dependent variables, operational definitions, validated assessment protocols, and appropriate analytical frameworks.

## Data Availability

All components and stages of this work were pre-registered through the Open Science Framework (10.17605/OSF.IO/TSZ7R). All associated files are available through its project website on osf.io/eqrax.

## Abbreviations

AFL: Australian Football League
ED: Ecological dynamics
RLD: Representative learning design
S&C: Strength & conditioning
